# Identifying important barriers to recruitment of patients in randomised clinical studies using a questionnaire for study personnel

**DOI:** 10.1186/s13063-019-3737-1

**Published:** 2019-10-30

**Authors:** Eva Isaksson, Per Wester, Ann Charlotte Laska, Per Näsman, Erik Lundström

**Affiliations:** 1 Department of Clinical Neuroscience, Neurology, Karolinska Institutet, Nobels väg 6, SE-171 76 Stockholm, Sweden; 2 Department of Clinical Sciences, Danderyd Hospital, Karolinska Institutet, SE-18288 Stockholm, Sweden; 3 Department of Public Health & Clinical Medicine, Umeå University, S-901 87 Umeå, Sweden; 4 Center for Safety Research, KTH Royal Institute of Technology, SE-100 44 Stockholm, Sweden; 5 Department of Clinical Neuroscience, Neurology, Uppsala University, SE-751 85 Uppsala, Sweden

**Keywords:** Recruitment, Survey, Questionnaire, Randomised controlled trials, RCT

## Abstract

**Background:**

Many randomised controlled trials (RCT) fail to meet their recruitment goals. Study personnel play a key role in recruitment. The aim of this study was to identify successful strategies that study personnel consider to be important in patient recruitment to RCT.

**Methods:**

We constructed a questionnaire based on the literature, discussions with colleagues and our own experience as trialists. The survey was named “What is Important for Making a Study Successful questionnaire” (WIMSS-q). Our target group was the study personnel in the ongoing EFFECTS study. The questionnaire was sent out electronically to all physicians and nurses (*n* = 148). Success factors and barriers were divided according to patient, centre and study level, respectively.

**Results:**

Responses were received from 94% of the study personnel (139/148). The five most important factors at centre level for enhancing recruitment were that the research question was important (97%), a simple procedure for providing information and gaining consent (92%), a highly engaged local principal investigator and research nurse (both 87%), and that study-related follow-ups are practically feasible and possible to coordinate with the clinical follow-up (87%). The most significant barrier at the local centre was lack of time and resources devoted to research (72%). Important patient-related barriers were fear of side effects (35%) and language problems (30%).

**Conclusions:**

For recruitment in an RCT to be successful, the research question must be relevant, and the protocol must be simple and easy to implement in the daily routine.

**Trial registration:**

The protocol for this study was registered at the Northern Ireland Hub for trials methodology research (SWAT ID 64). The EFFECTS study has EudraCT number 2011–006130-16 and was registered 17 February 2016 at ClinicalTrials.gov number NCT02683213.

## Background

### Introduction

A common problem with randomised controlled trials (RCT) is that the recruitment is slow and fails to meet the recruitment goal in time [1]. In 2007 Campbell found that less than one-third of trials achieved their original recruitment target goal in time [2]. Poor recruitment can lead to underpowered study results. It can also have practical, financial and ethical consequences, as it may prolong the trial. Furthermore, the study subjects consent to participate in a trial to help answer an important health question when, in fact, the question is not answered due to insufficient power.

Clearly there is a need for more research in this area [3, 4] and in the UK finding methods to enhance recruitment in RCTs has been identified as being of the highest priority [5, 6]. It is considered important to try to predict early on in the planning phase of a study what problems are likely to arise when it comes to the recruitment of patients [7]. Study personnel at centres play a crucial role in recruiting and retaining patients in clinical trials. Little is known about what people involved in clinical trials think are relevant barriers and solutions [8, 9]. Workshop and in-depth interview studies have investigated barriers to and opportunities for recruitment in clinical studies and found that it is still a challenge; new recruitment interventions are needed [10, 11].

Kaur et al. [12] were the first to develop a survey tool to capture the recruitment experience of clinical teams regarding facilitators and barriers to recruitment in clinical trials. The authors identified six important categories: (i) trial, (ii) site, (iii) patient, (iv) clinical team, (v) information and consent, and (vi) the study team.

## Aim

The aim of this study was to identify successful strategies that study personnel consider to be important for patient recruitment to RCT.

## Methods

The questionnaire was developed in several steps. First, we performed a literature search (February 2017) in PubMed using the following search terms: survey OR questionnaire OR recruitment challenges OR randomised controlled trial. We searched for original studies and systematic reviews in English. In addition to the literature search, we found information at the Trial Forge initiative [13, 14].

Secondly, we discussed this topic with colleagues from Edinburgh University (acknowledged below) with significant experience of conducting multicentre RCTs.

Finally, we used our own experience of conducting multicentre clinical trials in Sweden.

### Construction of the questionnaire

**Fig. 1:**
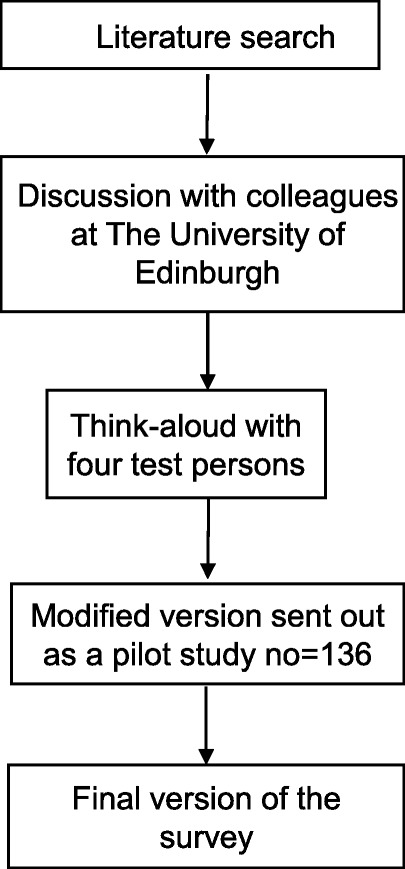
The process of developing the questionnaire

Figure 1 presents an overview of how we constructed the questionnaire. We decided to divide the questionnaire into success factors and barriers to recruitment in an RCT at patient, centre and study level, respectively.

In March 2017, we pre-tested the questionnaire using a think-aloud procedure [15]. One researcher (EI) sat side by side with the test person and wrote down all their comments while they performed the questionnaire. Four test persons with varying experience of working with clinical trials were chosen: one medical doctor with 25 years of experience with trials in neurology, one research nurse with 20 years of experience of research, one nurse with a PhD who had worked with research for 30 years, and the fourth was a nurse who had worked for 6 months as a research nurse. The questions were adapted based on what emerged from the pre-test; it appeared to be difficult for the participants to formulate five free-response alternatives indicating significant barriers and factors important for inclusion, so we reduced the number of alternatives to two. Further, some questions were reformulated. We named the questionnaire “What is Important for Making a Study Successful questionnaire” (WIMSS-q).

### Pilot study

To test the method, questions and the response rate, a pilot study was carried out March 2017. The questionnaire was sent electronically by SurveyMonkey [16] to all physicians and nurses active, listed in the delegation log at the time (*n* = 136) in Efficacy oF Fluoxetine—a randomised Controlled Trial in Stroke (EFFECTS) [17], an ongoing RCT in Sweden. We had a 67% response rate (92/136) after three reminders. The questionnaire was adjusted according to what was found (Additional file 1, Changes made in the questionnaire). In summary, we rephrased some of the questions and grouped them differently and removed the link to the EFFECTS study, which meant that the responders should answer the questionnaire with all their accumulated knowledge and experience of working with RCTs.

### Description of the final version of the questionnaire

WIMSS-q began with some general questions: age, gender, the role in EFFECTS, and how accustomed the participants were taking part in RCTs.

The general questions were followed by two open questions about barriers and important factors for inclusion in RCT. The purpose of these questions was to capture what we may have missed when we constructed the questionnaire.

**Fig. 2:**
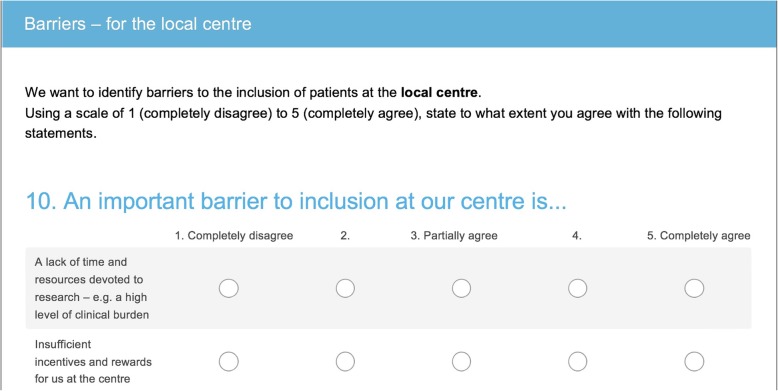
A question in the questionnaire

After these open questions, we asked questions with predefined response options. We provided a preformed list of potential factors affecting recruitment as well as facilitators for three levels: patient, local centre- and study-related barriers (Additional file 2, final version of the questionnaire). We used a five-point Likert scale [18, 19] ranging from 1 (completely disagree) to 5 (completely agree). One of the questions is illustrated in Fig. 2.

There were questions about ways to succeed with inclusion. We stated 16 factors and the participants were asked to grade them by using the Likert scale, 1 to 5. The questionnaire also had questions about differences between academic-driven studies and industry-financed studies and whether co-authorship or involvement in a sub-trial in both academic and industry-initiated studies was likely to influence the recruitment rate. Academic-driven studies are initiated, led and conducted by investigators from universities or hospitals, and no pharmaceutical company is involved in the trial. The survey ended with a question about the importance of communication about the trial in social media: web-sites, digital newsletters, twitter, Facebook, YouTube, Instagram and blogs.

### Main phase

In January 2018, the final WIMSS-q (Additional file 2) was sent electronically by SurveyMonkey, an online survey tool, to all physicians and nurses who were actively involved in the EFFECTS trial, i.e., those listed in the delegation log at the time (*N* = 148). It was largely speaking the same group of people that had previously participated in the pilot, with the addition of a few who became active in the study after the pilot phase and the removal of a few since they had stopped working in the study before the final version of the questionnaire was sent out.

They were instructed to answer using all their accumulated knowledge and experience of RCT; the questions were not specific to the EFFECTS trial. Participation in the survey was voluntary. None of the questions required mandatory answers. We informed the participant on the first page of the questionnaire that it was voluntary and that their decision would not influence our contact with them; if they said no we did not send any reminders.

Everyone who participated was given financial compensation in the form of a cinema voucher (worth approximately 11 Euros).

We sent three consecutive reminders to non-responders within a three-week period using the SurveyMonkey system and an additional ten reminders to the non-responders within a period of five weeks. In addition to that, we sent a personal email to non-responders.

### Number of questionnaires, data analyses and statistical methods

We had a 94% (139/148) response rate. The WIMSS-q took on average 13 min to complete.

The proportion of missing data within the questionnaires was low, below 3%. All data were exported from SurveyMonkey and entered into the SAS system from the SAS Institute (Cary, NC, USA) and descriptive statistics and graphical methods have been used to characterise the data.

## Results

**Fig. 3:**
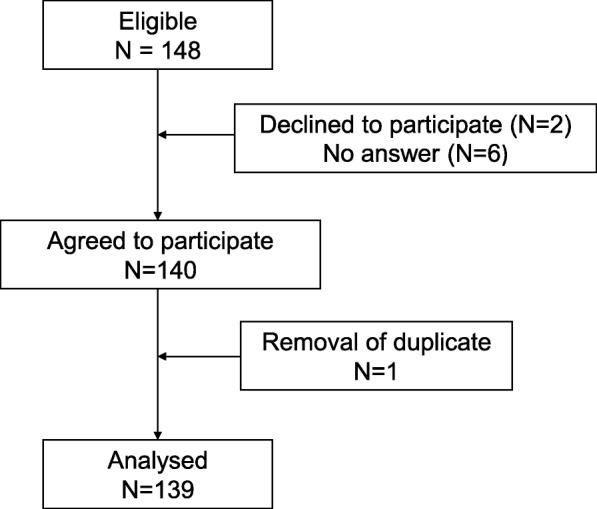
participant flow diagram

Of 139 responders, 71% were women and 29% men (Table 1). Their mean age was 47 years (SD 11 years). There were 53% physicians and 47% nurses. Two responders (1%) did not state their occupation. Figure 3 is the participant flow diagram.

**Table 1:** Baseline characteristics of the participants

Characteristics	*n* = 139
Age, year, mean (SD)	47 years (11 years)
Female n (%)	98 (71%)
Physicians, n (%)	72 (53%)
Nurses, n (%)	65 (47%)
Experience of clinical trials	
Very experienced*	13 (9%)
Quite experienced**	34 (25%)
Very inexperienced***	91 (66%)
Type of centre	
Acute stroke unit	115 (84%)
Neurorehabilitation unit	14 (10%)
Geriatric rehabilitation	8 (6%)

*Very inexperienced, EFFECTS was their first trial

**Quite experienced. Involvement in two or three studies during the past 5 years

***Very experienced. Involvement in five or more studies during the past 5 years or conducted their own research

Sixty-six percent were not accustomed to working with clinical trials; EFFECTS was the first trial in which they have participated (Table 1). Nine percent were very experienced, which means that they had been involved in five or more trials or had carried out their own research. Most of the participants worked at an acute stroke unit (84%).

### To succeed with inclusions in trials

The ways of ensuring that recruitment can meet its target are shown in Table 2. The most important factor is that the study personnel consider the research question being studied is relevant (97%). A simple consent procedure is also of importance (92%). Another highly valued factor is that the local principal investigator and the research nurse are highly engaged (87%) and that study-related follow-ups are simple and can be coordinated with the clinical follow-up (87%).

**Table 2:** To succeed with inclusion in a randomised controlled trial

Factors in questionnaire	n	%
Relevant question	124	97
Consent procedure is simple	118	92
Support team responds quickly to any questions	113	89
Local principal investigator is highly engaged	112	87
Research nurse is highly engaged	111	87
Follow-ups are simple and can be coordinated with the clinical follow-up	111	87
Involvement in the trial is fun	107	84
Regular contact between the main centre and the local centre	102	80
Those leading the trial are very enthusiastic	101	79
Regular information, digital newsletters or website updates	85	66
Regular investigation meetings	67	53
Regular nursing meetings	65	51
The trial is academic-driven	63	50
My centre includes many patients or participants in the trial	50	39
Basic compensation (e.g. cinema voucher)	44	34
Well-known researchers in the steering committee	27	21

n denotes the number of alternatives 4 and 5 on a Likert scale: 1 (completely disagree), 3 (partially agree) and 5 (completely agree)

Other important factors are that the central team leading the trial is very enthusiastic (79%), and that the support team responds quickly to questions (89%) and that those leading the trial provide regular information about what is happening in the trial by means of digital newsletters or website updates (66%).

Eighty-four percent said that it was important that involvement in the trial is fun. Intentionally, we did not specify what fun was. Each participant had to interpret the question based on their own experience.

It is of less importance that the steering committee consists of well-known researchers (21%) or that one’s own centre contributes many patients or participants to the trial (39%). Fifty-three percent thought that it was important that there are regular investigation meetings and 51% wanted meetings with only research nurses.

Fifty percent agreed that it was relevant in some way to have co-authorship in a scientific article and that it would influence their dedication to the trial. Of these, 10% said that this would influence them very much.

Fifty percent agreed that the opportunity to propose an idea for a sub-trial or an article once the main trial has been completed would influence their inclusion of individuals. Among them, 23% agreed a lot or completely agreed (4 or 5 according to the Lickert scale).

#### Patient-related barriers

The most important patient-related barrier for inclusion according to the study personnel shown in Table 3 was fear of side effects of the trial drug (35%). The second most important barrier was that the patient had language problems, aphasia/dysarthria (30%). Twenty percent mentioned that they thought that the patients had difficulties in understanding the importance and need for randomisation.

**Table 3:** Patient-related barriers

Factors in questionnaire	n	%
Fear of side effects	45	35
Language problems	39	30
Difficulties in understanding the importance of randomising	25	20
A fear of not receiving the best possible treatment	15	12

n denotes the number of alternatives 4 and 5 on a Likert scale: 1 (completely disagree), 3 (partially agree) and 5 (completely agree)

### Centre-related barriers

Table 4 shows the most important centre-related barriers to recruitment. Lack of time and resources devoted to research at the clinic (72%) were the most important. The absence of a research nurse was also an important factor (31%). A few thought that insufficient training in Good Clinical Practice affected inclusion in trials (5%) or feared that participation in the trial might harm the patient (3%). Insufficient financial compensation was not an important factor (5%).

**Table 4:** Centre-related barriers

Factors in questionnaire	n	%
Lack of time and resources	92	72
Absence of a research nurse	40	31
Lack of experience and organisation of research	22	17
Absence of a local principal investigator	15	12
Insufficient incentives and rewards	14	11
Competing trials	11	9
Insufficient training in the trial-specific instruments	7	5
Insufficient financial compensation	7	5
Insufficient training in GCP	6	5
Concern that participation in the trial might harm the patient	4	3

n denotes the number of alternatives 4 and 5 on a Likert scale: 1 (completely disagree), 3 (partially agree) and 5 (completely agree)

### Study-related barriers

Study-related barriers and complicating factors for inclusion of patients were: narrowly defined criteria for inclusion and exclusion (31%), complex and lengthy procedures for inclusion (17%) and comprehensive monitoring (12%) (Table 5).

**Table 5:** Study-related barriers

Factors in questionnaire	n	%
Narrowly defined criteria for inclusion and exclusion	40	31
That inclusion is not a simple process	22	17
Comprehensive monitoring	16	12
Weak and unclear organisation by those leading the trial	14	11
The regulations for clinical trials	10	8

n denotes the number of alternatives 4 and 5 on a Likert scale: 1 (completely disagree), 3 (partially agree) and 5 (completely agree)

Regarding social media, the most important factor was the presence of a study-specific website (79%) where you can provide information about the study, give access to essential documents and study specific tools such as the randomisation system.

Weekly digital web-letters from the central team were found to be of importance (51%). Twitter, Instagram, blog, YouTube or Facebook were not stated as being important.

Offering substantial financial compensation to the study team at the clinical and the university departments is more important in the case of an industry-funded study (73%) than that of an academic-driven study (45%).

In the two open questions about barriers and important measures for inclusion in RCTs at the beginning of the questionnaire we found that the screening procedure to find possible subjects for the trial was an important issue. Many stated that having a structured and organised screening process is the key to success.

## Discussion

In our study we found several factors that can be used by researchers running RCTs to improve the recruitment of patients. The most important factor for recruitment was that the study personnel consider the research question that is being studied to be relevant, which is in agreement with previous reports [20]. This might seem obvious, but it is important to have a strong “why” for people to engage in a study. Having a dedicated local principal investigator and a committed research nurse is a prerequisite [21–23].

In the two open questions, many of the respondents stated that an organised screening process to identify patients is vital. This was something that the personnel found challenging in everyday clinical activities. Our own experience as trialists is that a structured and organised screening process is the key to success and you should have time in the daily routine to do this [10, 24].

When planning a study, it is important to understand what the recruitment difficulties can be and then preferably implement strategies before the problems arise. Donovan et al. [7] interviewed trialists, study personnel and patients in the early phase of a study to learn about recruitment difficulties and made subsequent changes to overcome problems. Doing this will ensure more efficient and effective recruitment. With this study we have made it clearer what the barriers for recruitment can be and that it is of importance to address the problems early in the process, when writing the protocol and planning the study.

Participants in our survey found it important that trials are simple and managed skilfully to support the local centres. Close contact and regular information from an experienced and flexible trial office team would seem to be important. These factors were also found in a similar study, a survey of a paediatric trial in the acute setting by Kaur et al. [25].

This can be achieved with weekly letters, emails and personal contact. The most commonly reported strategies to improve recruitment were newsletters, mail, regular visits and phone calls. The use of posters or placards at the clinic for patients, relatives and staff reminding them of the study can be one way to do this. In centres with inexperienced personnel this is even more important. It is imperative that those who lead the study consider site-specific issues and work individually with each site based on what their requirements are [26, 27].

Our study found some of the keys to reducing waste in future studies and simplifying research-related procedures both for recruitment and follow-up [28]. The informed consent process must be comprehensible, which is especially important when it is the patient or the next of kin who make the decision, the entry criteria should be adjusted and appropriate to the group one intends to study, and it should be possible to coordinate these follow-ups with the clinical follow-up. Research must be integrated into the day-to-day work of the clinic [29].

The budget for a study, irrespective of whether it is academic or industry driven, must be large enough to allow activities such as trial meetings, training in trial-specific topics and participation in congresses. It is important to have a stable budget to achieve this. Also, small things such as shortbread or a cinema voucher can encourage study staff to go the extra mile as well as prioritise time for the study [30, 31].

The survey among the study personnel identified many barriers but also several promising methods by which to approach recruitment problems. As an example, the protocol and informed consent process should be simple and the study-related follow-up should be coordinated with the clinical follow-up. They also highlighted the importance of the availability and encouraging support of the central team in the event of questions.

The literature search made it clear that most of these strategies have never been the subject of research. We chose to focus on what is important for recruitment and have not investigated how to retain patients in studies, obtain compliance with a study drug or data quality.

The result of our WIMSS-q study could probably be generalised to other similar trials even outside the stroke area. We believe a relevant research question, a simple protocol and that it is easy to implement research in the daily clinical routine applies for all studies. There are, however, instances where these findings may not directly apply, for example, in patients with stroke which affects the brain and entails specific problems such as aphasia and fatigue, or other diseases, e.g., terminal pancreatic cancer, has its own recruitment problems that our survey certainly does not cover.

The study will add to knowledge about recruitment and management of RCTs. When planning a study, trialists should include recruitment strategies and evaluation of how they proceed in their trials. Finding which strategies are effective would be beneficial to the research community and to the society [32]. Treweek et al. state in their Cochrane analysis that rather than developing and testing new strategies, the evidence base should be improved by replicating evaluations of existing strategies [20].

The strength of this study is that inexperienced personnel have expressed their thoughts about participating in an RCT. We found that if you design pragmatic studies, they can also be performed advantageously in inexperienced centres.

Our study has some limitations that may have influenced the results obtained. The people who responded to the survey form part of a network with links to those of us who are responsible for the survey. This may have influenced both the response rate and the content of the responses. Another possible confounding factor is that EFFECTS is a study with broad criteria and simple protocols that can also affect the answers. Many in the study are unaccustomed to studies, this being the first study they have ever worked with. This can thus affect the results of the study. Unfortunately, we were not aware of the article of Kaur et al. before developing our questionnaire. This oversight may have affected the design. Interestingly, it turned out that our thoughts were similar, which can be interpreted as we have drawn the same conclusions about what is important when recruiting patients in studies.

Further, if we had combined a mixed model design we would probably have gained deeper knowledge [33].

This study may not be generalised to all RCTs, but for academic-driven RCTs its relevance and simplicity is of significance.

## Conclusions

For recruitment in an RCT to be successful, the research question must be relevant, and the protocol must be simple and easy to implement within the daily routine.

### Trial registration

We registered the protocol at the Northern Ireland Hub for trials methodology research [34].

## Supplementary information

**Additional file 1.** Changes in the questionnaire after the think-aloud and the pilot phase.

**Additional file 2.** Final version of the questionnaire.

## Data Availability

The dataset for WIMSS-q will be made available by the corresponding authors on reasonable request.

## Abbreviations

EFFECTS: Efficacy oF Fluoxetine – a randomisEd Controlled Trial in Stroke
RCT: Randomised controlled trial
WIMSS-q: What is Important for Making a Study Successful questionnaire
