# Supplementary material for: Identifying important barriers to recruitment of patients in randomised clinical studies using a questionnaire for study personnel
Source: Trials. 2019 Oct 30;20:618. doi: 10.1186/s13063-019-3737-1 (PMC6822437; doi:10.1186/s13063-019-3737-1)
Supplement: Supplementary file 1 — Additional file 1. Changes in the questionnaire after the think-aloud and the pilot phase. [file 13063_2019_3737_MOESM1_ESM.docx]

Additional file 1

**Changes in the questionnaire after the think-aloud and the pilot phase**

After testing the questionnaire using think-aloud and after the pilot phase we changed the introduction of the survey and asked the participants to answer the questionnaire based on their combined experience of research and not only from their experience of working with the EFFECTS- trial.

**Question 5, 6, 7 and 8**

When carrying out the think-aloud procedure we realised that it was hard for the participants to come up with 5 alternatives, so we reduced the number of alternatives to 2 for the following questions:

We would like you to state the two most important barriers to inclusion in randomised clinical trials. (Previous wording: we would like you to state the five most important barriers to inclusion in randomised clinical trials).

5. The most important barrier to inclusion in clinical trials is: (2 options instead of 5).

6. The second most important barrier to inclusion in clinical trials is: (2 options instead of 5).

What do you consider to be the two most important measures that would increase inclusion in randomised clinical trials? (previous wording: What do you consider to be the five most important measures that would increase inclusion in randomised clinical trials?).

7. The most important measure to increase inclusion in randomised clinical trials is: (2 options instead of 5).

8. The second most important measure to increase inclusion in randomised clinical trials is: (2 options instead of 5).

**Question 9**

We removed alternative 4: Fear of serious interactions between the study drug and regular drugs. We thought that this was difficult to answer since patients do not always understand the meaning of interactions between drugs.

**Question 10**

In the following text bolded denotes added text. These changes were made after the pilot phase:

Question 10 was rephrased, and the bolded text was added to make the wording clearer:

- an important barrier to inclusion **for a randomised clinical study** at our centre is...
- alternative 1 A **lack of time and resources devoted to research – e.g.** **a** high level of clinical burden.
- Alternative 3: Weak and unclear organisation **by those leading the trial.**

An alternative was removed because it was hard to understand the meaning for those not accustomed working with trials: unrealistic goals for the number of patients included at our centre.

**Question 12**

We merged 2 alternatives to 1 since they were considerably similar

- Alternative 1: “patient information is easy to understand” and alternative 2: “It is easy to carry out the consent procedure” was merged to: **Patient information and the consent procedure are simple.**
- Alternative 3: “The follow up is simple” and 4 “The follow up is coordinated with the clinical follow-up of the patients” were combined to: **Follow ups are simple and coordinated with the clinical follow-up.**

**Question 14, 15**

Changes were made since we thought that it was easier to choose a number by using a continuous scale meaning that the participants stated the importance using a scale by moving a circle between 0 (unimportant) and 100 (very important).

W removed:

- alternative 1: If the study is academically driven, then it is important to get substantial financial compensation
- alternative 2: If the study is industrially financed, then it is important to get substantial financial compensation.

These two alternatives were converted using a continuous scale and became question 14 and 15:

- 14. How important is substantial financial compensation if the trial is academic-driven? State the importance using the scale below by moving the circle between 0 (unimportant) and 100 (very important).
- 15. How important is substantial financial compensation if the trial is industry-financed? State the importance using the scale below by moving the circle between 0 (unimportant) and 100 (very important).

**Question 16, 17**

After discussions at an investigator´s meeting suggesting that co-authorship might increase the recruitment we added two questions:

Question 16. If you were offered co-authorship of a scientific article, would this influence your inclusion of individuals in a clinical research trial?

- No
- Yes, a little
- Yes, quite a lot
- Yes, very much so
- I have no opinion

Question 17. If you had the opportunity to propose an idea for a sub-trial or an article once the main trial has been completed, would this influence your inclusion of individuals in a clinical research trial?

- No
- Yes, a little
- Yes, quite a lot
- Yes, very much so
- I have no opinion

**Removed one question and rephrased question 13**

We removed the following question because we thought it was too long and unclear and difficult to answer:

We wonder how often you think you should have investigators´ meetings? By investigators we mean those who are part of the local team at each centre, i.e. all doctors and nurses who participate and do study-related tasks. How often should you conduct trial meetings for the entire study?

- Never
- Some one time per study i.e. when the study starts and when it ends
- Once a year
- Twice a year
- Three or more times a year

The removed question above was rephrased and moved to question 13:

13. To succeed with inclusion, it is important that...

- There are regular investigation meetings
- There are regular nursing meetings

We used a five-point Likert scale varying from 1 (completely disagree) to 5 (completely agree).
