# Supplementary material for: Identifying important barriers to recruitment of patients in randomised clinical studies using a questionnaire for study personnel
Source: Trials. 2019 Oct 30;20:618. doi: 10.1186/s13063-019-3737-1 (PMC6822437; doi:10.1186/s13063-019-3737-1)
Supplement: Supplementary file 2 — Additional file 2. Final version of the questionnaire. [file 13063_2019_3737_MOESM2_ESM.pdf]

What is important when recruiting for randomised trials?

Questionnaire about inclusion in trials

Hello,
This questionnaire aims to identify what you, as a doctor or nurse, consider to be important in the conducting of research trials. It is being sent to everyone who is actively involved in the EFFECTS trial.

The questionnaire is part of Eva Isaksson's doctoral project. It is intended to identify the factors that influence the recruitment of patients and will attempt to propose strategies that will be of benefit for future trials. Anonymised results will be published in a scientific journal. We have obtained ethical permission (**date and registration number**) to conduct this study.

In randomised clinical trials, it is often difficult to recruit patients within the planned time frame. This can result in the trial being extended and perhaps never reaching its objectives, which means that the question being investigated cannot be answered with any degree of statistical certainty.

When answering the questions, you must draw upon all your accumulated knowledge and experience of randomised clinical trials. The questions are not specific to EFFECTS.

Participation in the study is, of course, entirely voluntary. Your decision whether or not to participate will not influence our contact with you. If you do not wish to take part, we would appreciate it if you state this in response to the first question. This will prevent you from being sent any reminders.

The questionnaire begins with some questions about you. These are followed by questions about potential barriers to inclusion. The questionnaire concludes with a few questions about ways to improve inclusion.

We estimate that the questionnaire will take approximately 15 minutes to complete. If we receive your response before (**DATE**), we will provide you with basic financial compensation in the form of a cinema voucher (worth approximately SEK 120). This will only be paid if all questions are answered.

Thank you for contributing to the community of knowledge and the EFFECTS trial.

Eva Isaksson	Erik Lundström
Trial Manager	Chief Investigator

1. Do you agree to respond to this questionnaire and for the responses to be anonymously published at group level?

- ○ Yes, I accept the conditions
- ○ No

What is important when recruiting for randomised trials?

Background

We would like to know a few things about your background.

2. How old are you?

3. Gender

- Male
- Female

4. We wonder how accustomed you are to taking part in clinical trials. We would like you to grade your response from 1 (very unaccustomed) to 5 (very accustomed).

How accustomed are you to taking part in clinical trials?

1. Very unaccustomed. EFFECTS is the first trial. in which I'm taking part.	2	3. Quite experienced. I have been involved in 2-3 studies during the past 5 years.	4	5. Very experienced. I have been involveding ≥ 5 studies during the past 5 years, or conduct my own research.
○	○	○	○	○

Barriers – the two most important

We would like you to state the two most important barriers to inclusion in randomised clinical trials.

5. The most important barrier to inclusion in clinical trials is:

6. The second most important barrier to inclusion in clinical trials is:

Increase inclusion – the two most important measures

What do you consider to be the two most important measures that would increase inclusion in randomised clinical trials?

7. The most important measure to increase inclusion in randomised clinical trials is:

8. The second most important measure to increase inclusion in randomised clinical trials is:

What is important when recruiting for randomised trials?

Barriers – patient-related

We want to identify **patient-related barriers** to inclusion.
Using a scale of 1 (completely disagree) to 5 (completely agree), state to what extent you agree with the following statements.

9. An important barrier to inclusion is that the patient has...

	1. Completely disagree	2.	3. Partially agree	4.	5. Completely agree
Language problems	○	○	○	○	○
A fear of the side-effects of the trial drug	○	○	○	○	○
A fear of not receiving the best possible treatment	○	○	○	○	○
Difficulties in understanding the importance of randomising	○	○	○	○	○

Barriers – for the local centre

We want to identify barriers to the inclusion of patients at the **local centre**.
Using a scale of 1 (completely disagree) to 5 (completely agree), state to what extent you agree with the following statements.

10. An important barrier to inclusion at our centre is...

	1. Completely disagree	2.	3. Partially agree	4.	5. Completely agree
A lack of time and resources devoted to research – e.g. a high level of clinical burden	○	○	○	○	○
Insufficient incentives and rewards for us at the centre	○	○	○	○	○
Insufficient training in the trial-specific instruments	○	○	○	○	○
Insufficient training in Good Clinical Practice (GCP)	○	○	○	○	○
Concern that participation in the trial may harm the patient	○	○	○	○	○
A lack of experience and organisation of research	○	○	○	○	○
The absence of a research nurse	○	○	○	○	○
The absence of a principal investigator	○	○	○	○	○
Insufficient financial compensation	○	○	○	○	○
Competing trials	○	○	○	○	○

Barriers – trial-related

We want to identify **trial-related** barriers to the inclusion of patients.
Using a scale of 1 (completely disagree) to 5 (completely agree), state to what extent you agree with the following statements.

11. An important barrier to the inclusion of patients is...

	1. Completely disagree	2.	3. Partially agree	4.	5. Completely agree
That inclusion is not a simple process	○	○	○	○	○
Narrowly defined criteria for inclusion and exclusion	○	○	○	○	○
Weak and unclear organisation of those leading the trial	○	○	○	○	○
The regulations for clinical trials	○	○	○	○	○
Comprehensive monitoring	○	○	○	○	○

What is important when recruiting for randomised trials?

Recruitment – what is important: part 1

We want to identify the best methods to increase inclusion.
Using a scale of 1 (completely disagree) to 5 (completely agree), state to what extent you agree with the following statements.

12. To succeed with inclusion, it is important that...

	1. Completely disagree	2.	3. Partially agree	4.	5. Completely agree
Patient information and the consent procedure are simple	○	○	○	○	○
Follow-ups are simple and coordinated with the clinical follow-up	○	○	○	○	○
The local principal investigator is highly engaged	○	○	○	○	○
There is a highly engaged research nurse	○	○	○	○	○
There is regular contact between the main centre (that is leading the trial) and the local centre	○	○	○	○	○
Those leading the trial are very enthusiastic	○	○	○	○	○
Involvement in the trial is fun	○	○	○	○	○

Recruitment – what is important: part 2

Using a scale of 1 (completely disagree) to 5 (completely agree), state to what extent you agree with the following statements.

13. To succeed with inclusion, it is important that...

	1. Completely disagree	2.	3. Partially agree	4.	5. Completely agree
The trial is academic-driven – i.e. the trial is conducted by the researchers themselves and no pharmaceutical company is involved	○	○	○	○	○
The support team responds quickly to any questions	○	○	○	○	○
The question being studied is relevant	○	○	○	○	○
Those leading the trial provide regular information about what is happening in the trial – e.g. via digital newsletters or website updates	○	○	○	○	○
There are regular investigation meetings	○	○	○	○	○
There are regular nursing meetings	○	○	○	○	○
Basic compensation (e.g. in the form of a cinema voucher) is paid to included patients several times during the year	○	○	○	○	○
My particular centre includes many individuals	○	○	○	○	○
The steering committee consists of well-known researchers	○	○	○	○	○

Financial compensation and authorship

Does financial compensation make any difference and is it important to receive co-authorship?

14. How important is substantial financial compensation if **the trial is academic-driven?** State the importance using the scale below by moving the circle between 0 (unimportant) and 100 (very important)

0 50 100

15. How important is substantial financial compensation if **the trial is industry-financed?** State the importance using the scale below by moving the circle between 0 (unimportant) and 100 (very important)

0 50 100

16. If you were offered co-authorship of a scientific article, would this influence your inclusion of individuals in a clinical research trial?

- No
- Yes, a little
- Yes, quite a lot
- Yes, very much so
- I have no opinion

17. If you had the opportunity to propose an idea for a sub-trial or an article once the main trial has been completed, would this influence your inclusion of individuals in a clinical research trial?

- No
- Yes, a little
- Yes, quite a lot
- Yes, very much so
- I have no opinion

Social media

We have a few questions about social media.

18. On a scale of 1 (not at all important) to 5 (very important), how important is it for a trial to have...

	1. Not at all important	2.	3. Quite important	4.	5. Very important
A website	○	○	○	○	○
Digital newsletters	○	○	○	○	○
Facebook	○	○	○	○	○
Twitter	○	○	○	○	○
Instagram	○	○	○	○	○
A blog	○	○	○	○	○
A YouTube channel	○	○	○	○	○

Enter your name and address in the box below if you would like to receive a cinema voucher as thanks for your participation. If you do not wish to receive one, you can leave the box empty.

Thank you for taking the time to complete this questionnaire,

Eva Isaksson	Erik Lundström
Trial Manager	Chief Investigator

19. Enter your name and address here:
